# The Role of cf-HPV DNA as an Innovative Biomarker for Predicting the Recurrence or Persistence of Cervical Cancer

**DOI:** 10.3390/v17030409

**Published:** 2025-03-13

**Authors:** Márcia Poinho, Laura L. M. S. Dias, Layane S. Pinheiro, Flávia Níniver O. Gomes, Heidy H. M. F. Rondon, Mikele P. de Oliveira, Jhonnatan S. Souza, Higino F. Figueiredo, Daniel L. Lira, José E. Levi, Valquíria C. A. Martins, Kátia L. Torres

**Affiliations:** 1 Programa de Pós-Graduação em Imunologia Básica e Aplicada—PPGIBA, Universidade Federal do Amazonas, Manaus 69080-900, AM, Brazil; flavianiniver94@gmail.com (F.N.O.G.); fheidyhalanna@yahoo.com (H.H.M.F.R.); mikelerpraia@gmail.com (M.P.d.O.); jhonnatan.souza@ufam.edu.br (J.S.S.); alvesvalquiria@yahoo.com.br (V.C.A.M.); katialuztorres@hotmail.com (K.L.T.); 2 Faculdade de Ciências Farmacêuticas—FCF, Universidade Federal do Amazonas, Manaus 69080-900, AM, Brazil; lauraluizamsd98@gmail.com (L.L.M.S.D.); layane.spinheiro22@gmail.com (L.S.P.); 3 Fundação Centro de Controle de Oncologia do Estado do Amazonas, Manaus 69040-010, AM, Brazil; higinofelipe@icloud.com (H.F.F.); daniellourencolira@hotmail.com (D.L.L.); 4 Instituto de Medicina Tropical de São Paulo, Faculdade de Medicina da USP, São Paulo 05403-000, SP, Brazil; dudilevi@usp.br; 5 Rede de Vigilância Genômica em Saúde, Otimização da Assistência e Pesquisa no Estado do Amazonas—REGESAM, Manaus 69040-010, AM, Brazil

**Keywords:** HPV, cell-free DNA, cervical cancer, recurrence, persistence, post-treatment surveillance, Amazonian population

## Abstract

Background: Cervical cancer is highly prevalent among women in Amazonas, Brazil, mainly due to low screening coverage, and is diagnosed at a late stage, which compromises the treatment efficacy and survival rates. After treatment, recurrence is frequent, and there are few follow-up options to detect it. This highlights the urgent need for less-invasive biomarkers to monitor affected patients. Methods: This study employed real-time PCR, targeting the E7 gene of HPV types 16 and 18 to analyze cell-free DNA from plasma samples from 39 cervical cancer patients treated at the Oncology Control Center Foundation in Amazonas, Brazil. Results: cf-HPV 16 DNA was detected in 54% of the samples before treatment. The socioeconomic and behavioral data showed that 46.2% of the patients had low educational levels, 77% reported having a low income, 79.5% experienced an early sexual activity onset, and 15.4% had never undergone cytological screening. Persistence or recurrence occurred in 30.8% of cases over 4–33 months of follow-up, with cf-HPV DNA detectable in 75% of these cases. Conclusions: cf-HPV DNA in plasma is a promising biomarker for post-treatment surveillance, facilitating the earlier detection of persistence/recurrence. Incorporating this biomarker into clinical protocols could enhance outcomes and survival, particularly in underserved regions like the Amazon, where the access to healthcare is limited.

## 1. Introduction

Cervical cancer (CC) stands out as the fourth most common malignant neoplasm among women globally, constituting a significant public health challenge, especially in developing countries. Annually, approximately 604,127 new cases and 341,831 deaths are recorded worldwide [1,2]. For the triennium 2023–2025, Brazil is estimated to annually register 17,010 new cases of cervical cancer, corresponding to an incidence of 15.38 age-adjusted cases per 100,000 women. In 2020, the country accounted for 6627 deaths due to this disease, resulting in a mortality rate of 6.12 deaths per 100,000 women. Excluding non-melanoma skin tumors, cervical cancer is the second most common type in the Brazilian Northern Region, with an incidence of 20.48 cases per 100,000 women. Specifically, in the state of Amazonas, the adjusted incidence rate for 2023 is 31.7 new cases per 100,000 women, while the adjusted mortality rate was 14.49 deaths per 100,000 women in 2020 [3]. Persistent infection by oncogenic Human Papillomavirus (HPV) is intrinsically linked to the etiology of CC.

In the state of Amazonas, the high morbidity and mortality associated with CC can be attributed to late diagnosis and delayed treatment initiation [4]. Factors such as geography, culture, and environmental challenges, along with insufficient hospital infrastructure, exacerbate the difficulty of accessing health services, especially for women residing in remote areas [5,6]. This reality contributes to the late detection of the disease, increasing the risk of diagnosis at advanced stages, which implies more aggressive treatments and reduces the chances of it being cured [7,8,9]. Cervical cancer screening in Brazil is a cytology-based program, performed at decentralized Basic Health Units (UBSs in the Portuguese acronym) and offered by the universal public health system (SUS in the Portuguese acronym). Recently, Brazilian guidelines are under review, with a change to screening based on HPV DNA detection [10] starting from 2025. Currently, there is also a global consensus that both the follow-up treatment of precursor lesions and cervical cancer should be adequately monitored by molecular tests to improve prognoses and the possibility of tolerated management [11].

In general, approximately 30% of the patients will experience treatment failure (persistence or recurrence) within a five-year survival rate. The risk of treatment failure increases when the diagnosis is made at a more advanced stage [12]. Persistence is defined as the presence of the disease in a period of less than or equal to six months after the end of treatment, and recurrence is defined as evidence of the disease after a period of more than six months since the end of treatment. Treatment failure can be characterized clinically—by using physical examination and/or imaging—or cyto/histopathologically with biopsies, which can often be difficult due to the stenosis of the vaginal canal resulting from radiotherapy [13,14]. All these methods are considered inefficient in anticipating recurrence.

Recent advances in scientific research have revealed the diagnostic of circulating cell-free DNA (cfDNA), found in the serum and plasma of cancer patients, opening new perspectives for diagnosis and monitoring the treatment response [15]. Circulating tumor DNA (ctDNA), a form of cfDNA in plasma, has been shown to harbor the same genetic alterations found in the solid tumor, allowing for the real-time interrogation of the tumor genomics by a non-invasive procedure. As all CCs are expected to contain HPV-DNA, this marker has been increasingly evaluated for its diagnostic and prognostic value in the clinical management of CC [16]. Systematic reviews and meta-analyses have shown the utility of cf-HPV DNA testing for the early detection of recurrence/persistence or for monitoring the treatment response in patients undergoing treatment for CC [17], as well as anogenital and oropharyngeal cancer [18,19,20].

HPV genotypes 16 and 18 are together responsible for approximately 90% of all CC cases and are, therefore, also the most studied [21]. This study aims to analyze the presence of cf-HPV 16 and 18 DNA in plasma as a predictive marker of the recurrence/persistence of the disease in the treatment follow-up of cervical cancer.

## 2. Materials and Methods

### 2.1. Population Study and Samples

Between August 2020 and September 2022, thirty-nine patients diagnosed with cervical cancer associated with HPV 16 or 18 DNA by the molecular genotyping of tumor tissue or cervicovaginal scrapes were invited to participate in a study conducted at the Oncology Control Center Foundation of the state of Amazonas (FCECON, Manaus, Brazil). Women with metastatic disease at diagnosis were not included. The follow-up of the participants was structured in four stages: phase zero (before the start of treatment), phase 1 (about six months after the start of treatment), phase 2 (about nine months after the start of treatment), and phase 3 (about 18 months after the start of treatment). Participants were categorized into two groups, A (IA to IIB) and B (IIIA to IVA), according to the International Federation of Gynecology and Obstetrics (FIGO) classification [22]. The enrolled patients signed an Informed Consent Form and completed a questionnaire that collected sociodemographic, clinical, and risk information on HPV infection. The recurrence or persistence of the disease was defined by the presence of specific signs and symptoms, such as persistent vaginal bleeding and abdominal pain, which were verified in the clinical records of the patients, including medical observations and the results of histopathological and imaging exams.

### 2.2. Biological Samples Collection and Processing

During the initial phase of the study, before treatment, the tissue collection was performed either at the patient’s first outpatient visit or during surgical procedures. Tumor tissue fragments, measuring approximately 3 to 5 mm, were collected and stored in dry plastic microtubes, free of DNase and RNase, and kept at −30 °C until processing. Simultaneously, a blood sample was collected in 5 mL PPT^TM^ (Plasma Preparation Tube—BD Vacutainer^®^, Franklin Lakes, NJ, USA) with gel, through a venous puncture. These samples were processed to obtain plasma by centrifugation at 1400–1600× *g* for ten minutes, no more than two hours after collection. The plasma was separated into aliquots and stored at −30 °C. To ensure the complete removal of cellular debris before DNA extraction, a second centrifugation at 16,000× *g* for ten minutes at 4 °C was performed. For cases without an indication for surgical treatment and tissue fragment collection, cervicovaginal material was collected by the medical doctor in the outpatient clinic using a Cervex-Brush^®^ Combi (Rovers^®^ Medical Devices B.V, Oss, The Netherlands). After the collection, the removable head of the brush was placed in a tube containing 4M guanidine thiocyanate preservative solution and vigorously shaken for 15 s, then the sample was separated into aliquots and stored at −30 °C. During the follow-up, blood samples were collected as the patients visited the hospital for any medical, dental, psychological, social service, or physiotherapy consultations or during home visits conducted by the FCECON social service.

Considering the greater clinical importance of HPV 16 and 18 infections and for reasons of technical feasibility, the molecular screening for the HPV 16 and 18 genotypes was initially conducted on tumor tissue samples or cervicovaginal material. Only the patients whose tissue samples were positive for HPV 16 and/or 18 DNA proceeded to the plasma analysis and the subsequent follow-up.

### 2.3. DNA Extraction

Tissue—DNA extraction from 20–40 mg of frozen tissues was performed using the DNeasy^®^ Blood & Tissue Kit (QIAGEN Inc., Valencia, CA, USA), following the manufacturer’s recommendations. The final volume of the extracted DNA was 200 µL, and the aliquot was stored at −30 °C. Plasma and cervicovaginal material—for the extraction of DNA from 800 µL of frozen plasma and cervicovaginal fluid, the ReliaPrep™ Blood gDNA Miniprep System (Promega Inc., Madison, WI, USA) was used, according to the manufacturer’s recommendations. The final volume of the extracted DNA from the plasma was 40 µL and from the cervicovaginal material was 60 µL. The extracted DNA was stored at −30 °C.

### 2.4. Human β-Actin PCR

To ensure the quality of DNA extraction, the human β-actin gene was amplified by a real-time polymerase chain reaction (qPCR) using Primer F (5′CCATCTACGAGGGGTATGC3′); Primer R (5′GGTGAGGATCTTCATGAGGTA3′); and a probe (5′VIC-CCTGCGTCTGGACCTGGCTG-NFQ3′) (Life Technologies, São Paulo, Brazil). The qPCR reaction was prepared with a final volume of 10 μL, containing 1× TaqMan master mix (Applied Biosystems, Foster City, CA, USA), 300 nM each of the forward and reverse primers, 100 nM of the TaqMan fluorogenic probe, and 50–100 ng of the DNA for the tissue samples. The DNA extracted from plasma could not be quantified, and the same volume of the extracted DNA applied to the tissue samples was used for the cf-DNA obtained from the plasma. The amplification protocol started at 50 °C for two minutes, followed by 95 °C for ten minutes, and continued with 40 cycles of 95 °C for 15 s, 55 °C for one minute, and 60 °C for one minute on the QuantStudio™ 5 system (Thermo Fisher Scientific© Inc., Waltham, MA, USA).

### 2.5. E7 HPV16/HPV18 Type-Specific Quantitative Real-Time PCR (qPCR)

All the samples were subjected to quantitative real-time PCR (qPCR) assays based on the TaqMan technology, targeting the E7 genes of HPV16 and HPV18. The assays and controls were performed in duplicate.

HPV16-E7—performed using the forward primer (5′GATGAAATAGATGGTCCAGC3′), the reverse primer (5′GCTTTGTACGCACAACCGAAGC3′), and the probe (5′FAM-CAAGCAGAACCGGACAG-MGB-NFQ) in a final reaction volume of 12.5 μL. The mixture for each qPCR reaction contained 1× TaqMan master mix (Applied Biosystems, Foster City, CA, USA), 300 nM each of the forward and the reverse primer, 100 nM of the TaqMan fluorogenic probe, and between 50 and 100 ng of the DNA for the tissue samples [23]. The cycling conditions were the same as above. As a positive control, 139 ng of DNA from the SiHa cell line, containing 1–2 copies of HPV 16 per cell, was used.

HPV18-E7—the assay included the forward primer (5′AAGAAAACGATGAAATAGATGGA3′), the reverse primer (5′GGCTTCCACCTTACAACACA3′), and the probe (5′VIC-AATCATCAACATTTACCAGCC-MGBNFQ3′) in a final volume of 12.5 μL. The composition of the qPCR reaction was the same as the assay for HPV16-E7. As a positive control, 75 ng of DNA from the HeLa cell line, containing 10–20 copies of HPV 18 per cell, was used. Purified DNA from the SiHa and HeLa cell lines were provided by one of the authors (JE Levi).

The HPV 16 viral load in the plasma sample—a standard curve was constructed using DNA extracted from SiHa cells (one copy of HPV 16 per cell) at a concentration of 55.6 ng/µL. Serial dilutions (1:10) were prepared to generate the standard curve, in accordance with the MIQE guidelines (Minimum Information for Publication of Quantitative real-time PCR Experiments) [24].

### 2.6. Statistical Analysis

The descriptive analyses were presented as absolute frequencies (*n*) and relative percentages (%), using tables and graphs. The detection of circulating HPV DNA in the plasma, before and during treatment, was visualized through Swimmer Plots, created in Microsoft Excel (version 2307, build 16626.20132) and Adobe Illustrator programs (version 26.5.0.223,2022). Survival was analyzed by the Kaplan–Meier method, with the comparisons between the groups performed by the Log-Rank test. The survival time was calculated from the start of treatment to either death or the last follow-up date. For the patients who did not start treatment, the date of inclusion in the study was considered the starting point. The analysis of the time until the start of treatment (Δt) was based on the interval from the first consultation to the start of treatment. The association between the clinical and histopathological characteristics of patients and the presence of markers during the follow-up was evaluated by the Pearson’s Chi-Square test, using the IBM SPSS Statistics software version 21, with a significance level of 5%.

## 3. Results

This study included 39 cervical cancer patients treated at FCECON from August 2020 to September 2022, divided into two groups according to FIGO staging: Group A (13 patients, FIGO IA to IIB) and Group B (26 patients, FIGO IIIA to IVA). The sociodemographic analysis revealed an age range of 25 to 80 years, with an average of 48.4 years (±13.4). About 46.2% of the participants had education up to incomplete elementary school and 77.0% had no income or received up to one minimum wage (<USD 286.00) (Table 1).

The risk factors for cervical cancer (CC) included the onset of sexual activity between 12 and 17 years (79.5%), having between two and five sexual partners (61.5%), and the occasional use of condoms (61.5%). In Brazil, screening for cervical cancer is carried out mainly through the Pap smear, recommended for women between 25 and 64 years old who have already started their sexual life. It was observed that 43.6% underwent more-frequent screening than recommended by the Brazilian guidelines, while 46.1% underwent fewer exams than indicated. Sexually transmitted infections were reported by 15.4% of patients, including one case of HIV (Table 2). Barriers to CC screening such as inhibition, shame, fear, or the lack of time were reported by 59.1% of the participants (Figure S1—Supplementary Material).

From a histological point of view, 89.7% of the diagnoses were squamous cell carcinoma and most (66.7%) were in advanced stages (III and IV), according to the FIGO classification. Most (74.4%) of the patients were treated with chemotherapy and radiotherapy, although three did not receive treatment due to various reasons, including an indication for palliative treatment and financial difficulties for transportation to the treatment unit (Table S1 Supplementary Material).

For this study, the clinical follow-up time varied from 1 to 33 months, depending on each patient (due to situations such as treatment abandonment, death, or the unavailability of the data). The therapeutic analysis showed that 12 patients (30.8%) presented the recurrence or persistence of the disease, and 4 (10.3%) died (Table 3). Of the 12 that presented recurrence or persistence, 1 (8.3%) was from Group A (FIGO (IA to IIB) and 11 (91.7%) were from Group B (FIGO IIIA to IVA).

The detection of HPV in the tissue showed that 39 samples were HPV 16 and 3 HPV 18 positive; in plasma 21/39 (53.8%) of the samples were positive for cf-HPV 16 DNA, no samples were cf-HPV 18 DNA positive, and 18 (46.2%) samples were cf-HPV 16 or 18 DNA negative at the beginning of the study (T_0_). In the follow-up, the proportion of patients with detectable circulating HPV DNA reduced to 16.7% after 18 months of treatment, without a statistically significant difference over time (Table 4).

The relationship between FIGO staging and the presence of cf-HPV DNA indicated that the patients in advanced stages had a higher frequency of detection (81.0%), with statistical significance (*p* = 0.041). Recurrence or persistence was observed in 33.3% of the women with detectable cf-HPV DNA, of which 19% died (Table 5).

**Figure 1:**
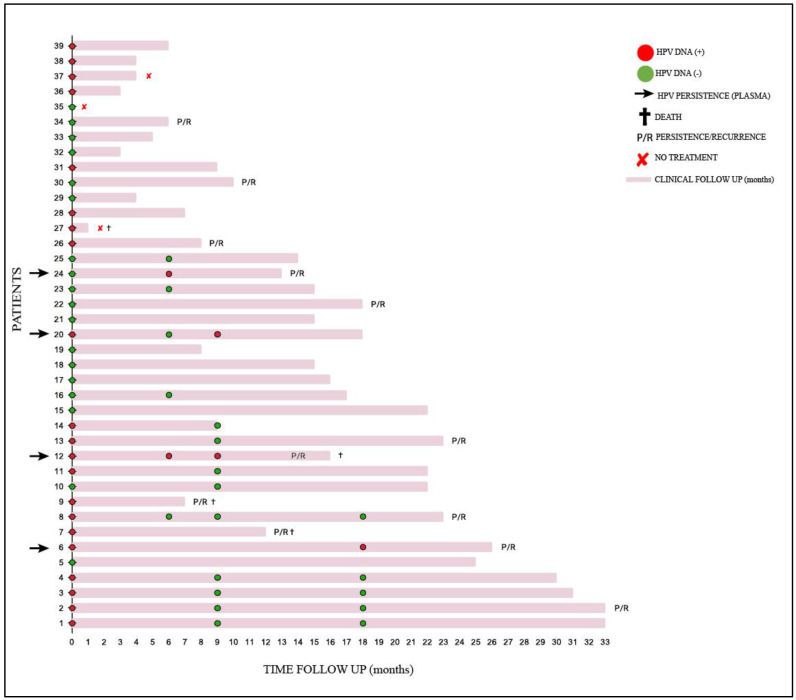
Clinical follow-up of 39 patients with cervical cancer treated at FCECON, from August 2020 to September 2022, Manaus—AM, Brazil. Each line corresponds to one patient (n = 39); marks in red (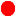) and green (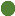) correspond to plasma samples positive for circulating HPV DNA and negative for circulating HPV DNA, respectively. The patients with detectable circulating HPV DNA in their samples during treatment are indicated by a black arrow; (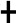) patients died; P/R patients presented persistence/recurrence; (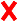) patients did not have treatment.

It was not possible to collect plasma from all the patients for the detection of cf-HPV DNA during the follow-up; 11 of the 16 monitored patients were positive for cf-HPV DNA at diagnosis, and four remained positive after treatment. In Table 6, detailed information is provided for the 12 cases that presented persistence/recurrence. The trajectory of the patients included in this study can be verified at Figure 1 (all 39 patients).

**Figure 2:**
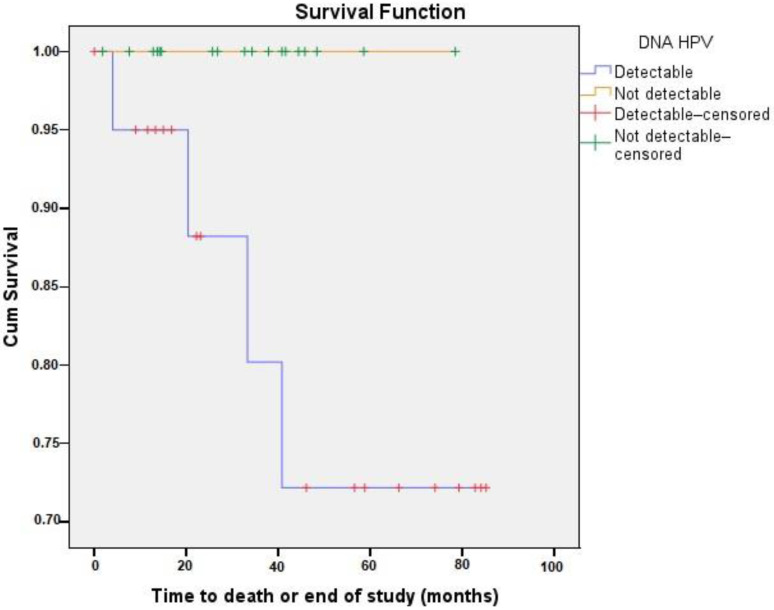
Survival analysis of the women (*n* = 39) monitored through cf-HPV DNA testing (cf-HPV DNA detectable in blue and not detectable in yellow) after the initiation of cervical cancer treatment at FCECON, Manaus, AM, Brazil, from August 2020 to September 2022.

The Kaplan–Meier survival curve suggested a trend of lower survival for the patients with detectable cf-HPV DNA at any time post-treatment, although the difference did not reach statistical significance (*p* = 0.073) (Figure 2). The interval between the first consultation and the start of treatment ranged from 1 to 29 months, with an average of 6 months (±5 months), indicating a varied distribution of time until treatment among patients.

## 4. Discussion

This study evaluated the use of cell-free HPV DNA (cf-HPV DNA) as a prognostic marker for disease persistence or recurrence in patients with cervical cancer after treatment. The research focused on patients from the Northern Region of Brazil, where the prevalence of cervical cancer is high, often exacerbated by difficulties in accessing diagnosis and treatment. Delays in the diagnosis and treatment of cervical cancer can be attributed to multiple factors, including the limitations of patients, health professionals, and the structure of health services [25,26]. The COVID-19 pandemic in 2020 exacerbated these challenges, impacting the screening and diagnosis of cervical lesions and highlighting the fragilities of the Brazilian health system, especially in the Northern Region, marked by pronounced social inequalities.

The state of Amazonas, with its vast geographical area (1,559,159,148 km^2^) and cultural, geospatial, and environmental challenges, exemplifies the difficulties in accessing health services. Only five health units in the SUS offer colposcopy exams, all located in the capital, Manaus, leaving women from the interior unassisted. It is important to mention that 71.5% of the Brazilian population depends on access to the public health service [27,28]. This scenario contributes to the late diagnosis of the disease, as observed in 26 (66.7%) of the patients in this study, classified in Group B (FIGO IIIA to IVA), with a prevalence of stage IIIC1 (23.1%), aligning with the literature, which reports diagnosis at advanced stages in about 50% of cases in Brazil [29].

This study revealed an average age of 48.4 years (±SD 13.4) among patients, quite similar to other studies [30]. However, it was notable that 35.9% of patients were between 21 and 40 years, reflecting similar findings by Moysés et al. [31] in 2019, who described a predominant age profile of between 35 and 39 years for cervical cancer in Manaus.

In this context, 46.2% of the women in this study reported being illiterate or having an incomplete elementary education. These data are corroborated by a previous study in Manaus, where 27.2% of patients treated for CC at FCECON presented with low educational levels [31]. Similarly, Lucena et al. [32], in a study conducted in Porto Velho, Rondônia (north of Brazil), found that 34.3% of women had little education, reinforcing the prevalence of a low educational level in the female population of the Northern Region of Brazil.

Screening for precursor lesions is challenging in the Amazon region due to geographical isolation, especially for women in riverside communities. This study revealed that 48.7% of the patients reported being from the interior of the state. However, when asked where they had lived in the last five years, 64.1% reported living in the capital. However, in some situations where contact with the patient was necessary, there were difficulties in finding them at the informed addresses.

Economically, 77.0% of women reported having no family income or an income of up to one minimum Brazilian wage (USD 286.00), underlining the influence of socioeconomic conditions on the performance of screening exams and on the incidence and mortality of the disease [33,34]. The early onset of sexual activity was observed as a risk factor for the development of CC, which aligned with studies showing an association between the early onset of sexual activity and a higher risk of cervical cancer [33,34,35]. This study also depicted the low adherence to the Pap smear test, with 68.2% of the women reporting barriers such as inhibition, shame, fear, a lack of time, or carelessness with health, emphasizing the importance of approaches that consider cultural and emotional barriers to screening [36,37,38,39,40].

Women living with HIV/acquired immunodeficiency syndrome (AIDS), especially those with immunosuppression, have a higher susceptibility to persistent infections by high-risk HPV types, increasing the risk of developing cervical dysplasia and cervical cancer [41]. This study identified one patient with HIV infection, an expected finding since HIV-positive women have four to five times more chances of developing cervical cancer (CC), as evidenced by Clifford et al. (2017) [42], who found a high prevalence of HPV types 16, 18, and 45 among HIV-infected women with invasive cervical cancer. Bowden et al. (2023) [43] also observed that HIV positivity decreases the likelihood of eliminating high-risk HPV.

In this study, squamous cell carcinoma (SCC) was the most common histological type (89.7%), followed by adenocarcinoma (ADC) (10.3%), aligning with findings by de Sanjose et al. (2010) [44], who analyzed cervical cancer samples from 38 countries and found a predominance of SCC. Other Brazilian studies corroborate these results, showing a similar prevalence of SCC in cervical cancer diagnoses [35,45,46]. Considering that approximately 35% of CC cases are diagnosed at advanced stages (III and IV) in Brazil [47], this research found a significant proportion of patients (23.1%) at an advanced stage (IIIC1), reflecting the influence of socioeconomic and demographic factors on accessibility to health services and, consequently, on the stage at which the disease is diagnosed [48].

Three patients (7.7%) did not receive treatment due to various reasons, including an indication for palliative care, a lack of financial resources for transportation, and the decision to return to their hometown. These findings highlight how a low socioeconomic status can prevent access to adequate information about CC and necessary treatment [49], leading to decisions that compromise the patient’s prognosis.

The detection of cf-HPV 16 DNA in plasma samples was observed in 21/39 (53.8%) of the patients at diagnosis (T_0_), while genotype 18 was not detected. These data are consistent with previous studies reporting variations in the prevalence of HPV genotypes [50,51,52].

In this study, persistence/recurrence was detected in 12/39 (30.8%) of the women studied, as described in the literature [12]. A systematic review revealed that most of the persistence/recurrence of the disease was detected within two to five years after the primary treatment for CC [53], as in our study, corroborating the importance of continuous monitoring for the early detection of recurrences.

Eighteen samples with undetectable cf-HPV DNA were found at (T_0_). Bonlokke et al. (2023) [54] conducted a study to investigate the diagnostic value of circulating HPV 16 and 18 DNA in patients with CC and identified that the amount of cf-HPV DNA is closely related to different clinical parameters such as the disease stage and tumor size. However, for patients with a lower disease burden, cf-HPV DNA positivity is significantly lower, i.e., a certain disease burden is required for cf-HPV DNA to be detected or even for it to be present in plasma. On the other hand, a meta-analysis was performed to evaluate the applications of cf-HPV DNA as a biomarker in cervical cancer, addressing its accuracy. It was reported that most studies employed the qPCR method, which demonstrated relatively high specificity but low sensitivity, since with qPCR it is difficult to detect very small amounts of nucleic acids circulating in the blood [16]. In this sense, the digital polymerase chain reaction (dPCR) may provide an alternative for molecular diagnosis, with greater sensitivity, accuracy, and specificity in relation to the qPCR method so that, if available, it can be used in these cases [55].

The SARS-CoV-2 pandemic posed significant challenges to women’s access to the Oncology Control Center Foundation (FCECON), directly affecting sampling and the continuity of biological sample collections as planned. This interruption resulted in an incomplete analysis of the four phases envisaged in the study, restricting the robustness of the analyses due to the limited sample size. Additionally, the consultation and interpretation of patient records, essential for identifying cases of the persistence or recurrence of the disease, were hindered by the inconsistency of the records made by the care team, with the absence of crucial information. Another limiting factor was the follow-up period ranging from 6 to 18 months, which may have affected the survival analysis of the patients.

Even with these important limitations, this study shows that the analysis of cf-HPV DNA can feasibly be performed in the region, providing oncologists with important information at a low cost and with simple sample collection, which may have a positive impact on CC patients from the Amazon.

## 5. Conclusions

Here, we demonstrate that the detection of cf-HPV DNA after treatment in patients with cervical cancer can serve as a warning for the development of the recurrence/persistence of the disease. Furthermore, the detection of cf-HPV DNA was able to identify, in one case, recurrence/persistence up to six months before the clinical diagnosis. However, more studies need to be conducted to standardize the tool as a routine biomarker in clinical practice.

## Figures and Tables

**Table 1:** Sociodemographic and economic characterization of patients with cervical cancer treated at FCECON from August 2020 to September 2022, Manaus—AM, Brazil.

Variables	*n* (39)	%
Age Range		
21–40	14	35.9
41–55	17	43.6
56–65	2	5.1
>65	6	15.4
Ethnic group		
White	3	77
Black	1	2.6
Brown	35	89.7
Education level		
Illiterate	6	15.4
Incomplete fundamental	12	30.8
Complete fundamental	1	2.6
Full high school	15	38.5
Incomplete higher education	1	2.6
Complete higher education	4	10.3
Marital status		
Single	14	35.9
Married	17	43.6
Divorced	2	5.1
Widow	6	15.4
Place of birth		
Capital (Manaus)	9	23.1
Interior of Amazonas state	19	48.7
Other States of Brazil	11	28.2
Residential history (last five years)		
Capital	25	64.1
Interior of Amazonas state	11	28.2
Other States of Brazil	3	7.7
Family income		
No economic income	15	38.5
Until 1 MW	15	38.5
2–3 MW	8	20.5
>3 MW	1	2.6

MW: minimum wage in Brazil (USD 286.00).

**Table 2:** Characterization of behavioral and risk factors of patients with cervical cancer treated at FCECON from August 2020 to September 2022, Manaus—AM, Brazil.

Variable	*n* (39)	%
Sexual debut (age)		
12–14	12	30.8
15–17	19	48.7
From 18 years old	7	17.9
Not informed	1	2.6
Sexual Partners		
Only 1	4	10.3
2–5	24	61.5
6–10	8	20.5
>10	1	2.6
Unknown	2	5.1
Condom Use		
Sometimes	24	61.5
Always	1	2.6
Never	14	35.9
Screening by cytology		
Every 6 months	3	7.7
Once per year	9	23.1
Twice in 2 years	5	12.8
Once in more than 3 years	12	30.7
Never	6	15.4
Not informed	4	10.3
History of smoking		
Yes	15	38.5
No	24	61.5
STI		
Yes	6	15.4
No	23	59.0
Do not know	10	25.6
Type of STI	*n* (6)	%
HIV	1	16.7
Syphilis	1	16.7
Unknown	4	66.7

STI: sexually transmitted infection; HIV: human immunodeficiency virus.

**Table 3:** Frequency of recurrence/persistence cases and clinical outcomes in patients with cervical cancer treated at FCECON from August 2020 to September 2022, Manaus—AM, Brazil.

Variables	*n* = 39	%
FIGO		%
Group A (IA to IIB)	13	33.3
Group B (IIIA to IVA)	26	66.7
Persistence/Recurrence		
Yes	12	30.8
No	24	61.5
No treatment	3	7.7
Outcome		
Death	4	10.3
Alive	35	89.7

FIGO: International Federation of Gynecology and Obstetrics.

**Table 4:** Frequency of cf-HPV 16 DNA positivity in patients with cervical cancer treated at FCECON from August 2020 to September 2022, Manaus—AM, Brazil.

cf-HPV16 DNA							
TIME	Patients (*n*)	%	Detectable	%	Undetectable	%	*p* *
T_0_	39	100	21	53.8	18	46.2	
T_1_	7	17.9	2	28.6	5	71.4	0.410
T_2_	11	28.2	2	18.2	9	81.8	0.046
T_3_	6	15.4	1	16.7	5	83.3	0.187

cf-HPV16 DNA: cell-free HPV16 DNA. * Significant *p*-value for *p* < 0.05 (5%).

**Table 5:** The relationship between the clinical characteristics of the patients with cf-HPV16 DNA, treated at FCECON from August 2020 to September 2022, Manaus—AM, Brazil.

Variables	cf-HPV16 DNA				*n* (39)	*p* *
	Detectable (*n* = 21)	%	Undetectable (*n* = 18)	%		
FIGO						0.041 *
Group A	4	19.0	9	50.0	13	
Group B	17	81.0	9	50.0	26	
Histology						
AC	2	9.5	2	11.1	4	0.636
SCC	19	90.5	16	88.9	35	
Persistence/Recurrence						
Yes	6	28.6	6	33.3	12	0.491
No	15	71.4	12	66.7	27	
Outcome						
Death	4	19.0	0	0.0	4	0.073
Survivor	17	81.0	18	100.0	35	

* Significant *p*-value for *p* < 0.05 (5%). Pearson’s Chi-Square test. cf-HPV16 DNA: cell-free HPV16 DNA; AC: adenocarcinoma; SCC: squamous cell carcinoma.

**Table 6:** Clinicopathological characteristics of 12 patients who presented persistence/recurrence.

Patients ID	Age (Years)	cf-HPV DNA Positive/Viral Load Copies/mL of Plasma (qPCR)				FIGO Staging	Histology	Death	Persistence/Recurrence Time Until Diagnosis (Months)
		T0	T1	T2	T3				
2	34	1.94	NSC	UD	UD	IIIC1	SCC	No	33
6	53	40.33	NSC	NSC	5.13	IVA	SCC	No	26
7	51	1.12	NSC	NSC	NSC	IVA	SCC	Yes	12
8	26	7.47	UD	UD	UD	IIIC2	SCC	No	23
9	48	486.23	NSC	NSC	NSC	IIIB	SCC	Yes	7
12	41	54.72	6.12	68.09	NSC	IIIC1	SCC	Yes	14
13	61	43.4	NSC	UD	NSC	IIIC1	SCC	No	23
22	38	UD	NSC	NSC	NSC	IIB	SCC	No	18
24	52	UD	0.92	NSC	NSC	IIIB	SCC	No	13
26	62	1.53	NSC	NSC	NSC	IIIC1	SCC	No	8
30	31	UD	NSC	NSC	NSC	IIIB	SCC	No	10
34	74	UD	NSC	NSC	NSC	III	SCC	No	6

cf-HPV 16 DNA: cell-free HPV 16 DNA; qPCR—quantitative polymerase chain reaction; T0—Time 0 (before starting treatment); T1—Time 1 (about 6 months after starting treatment); T2—Time 2 (about 9 months after starting treatment); T3—Time 3 (about 18 months after starting treatment); UD—undetectable; FIGO—International Federation of Gynecology and Obstetrics; NSC—sample not collected; SCC—squamous cell carcinoma.

## Data Availability

The original contributions presented in the study are included in the article/Supplementary Material; further inquiries can be directed to the corresponding author.

## Supplementary Materials

The following supporting information can be downloaded at https://www.mdpi.com/article/10.3390/v17030409/s1, Figure S1. Reasons why patients with cervical cancer, treated at FCECON from August 2020 to September 2022, Manaus, AM, Brazil, did not routinely perform preventive care (n = 22); Table S1. Clinical characterization of patients with cervical cancer treated at FCECON from August 2020 to September 2022, Manaus—AM, Brazil.
