# Supplementary material for: The Role of cf-HPV DNA as an Innovative Biomarker for Predicting the Recurrence or Persistence of Cervical Cancer
Source: Viruses. 2025 Mar 13;17(3):409. doi: 10.3390/v17030409 (PMC11946245; doi:10.3390/v17030409)
Supplement: Supplementary file 1 [file viruses-17-00409-s001.zip › viruses-3374061-supplementary.pdf]

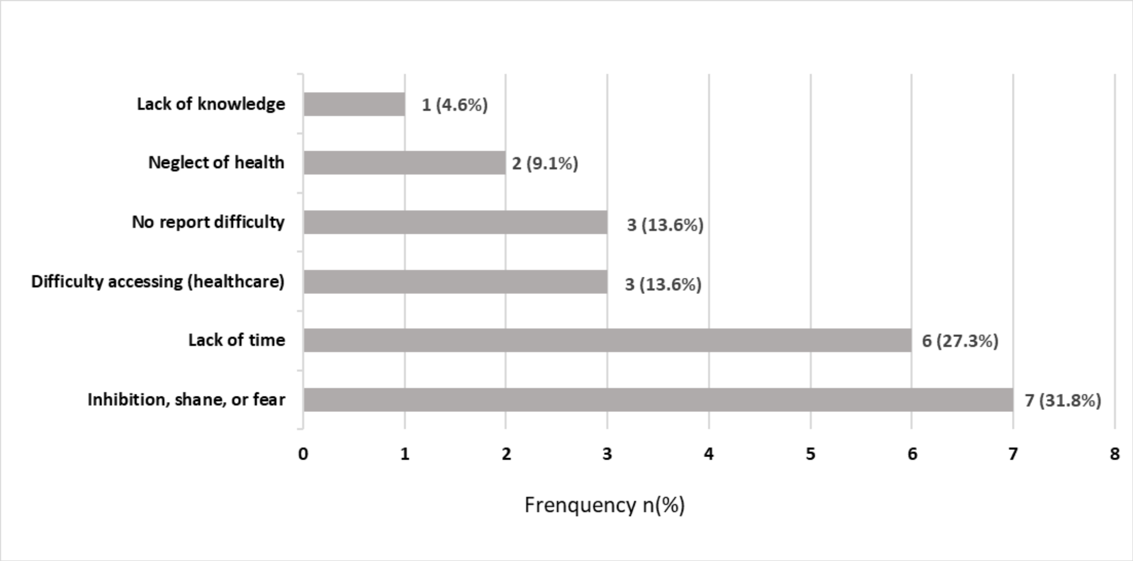

Figure S1. Reason why patients with cervical cancer, treated at FCECON from August 2020 to September 2022, Manaus – AM, Brazil, did not routinely perform preventive care (*n*=22)

Table S1. Clinical characterization of patients with cervical cancer treated at FCECON, from August 2020 to September 2022, Manaus – AM, Brazil.

Variables	*n* (39)	%
Histology		
AC	4	10.3
SCC	35	89.7
FIGO stage		
IA2	1	2.6
IB1	3	7.7
IB2	2	5.1
II	1	2.6
IIA	1	2.6
IIB	4	10.3
IIC1	1	2.6
III	5	12.8
IIIB	6	15.4
IIIC	1	2.6
IIIC1	9	23.1
IIIC2	2	5.1
IVA	3	7.7
Treatment		
Surgery	7	17.9
Chemo + RTx	29	74.4
No treatment	3	7.7

AC: Adenocarcinoma; SCC: Squamous Cell Carcinoma; Chemo: Chemotherapy; RTx: Radiotherapy; FIGO: International Federation of Gynecology and Obstetrics
